# Hearing Thresholds Changes after MRI 1.5T of Head and Neck

**DOI:** 10.1155/2019/8756579

**Published:** 2019-06-17

**Authors:** Maryam Bahaloo, Mohammad Hossein Davari, Mohammad Sobhan, Seyyed Jalil Mirmohammadi, Mohammad Taghi Jalalian, Mohammad Javad Zare Sakhvidi, Farimah Shamsi, Sam Mirfendereski, Abolfazl Mollasadeghi, Amir Houshang Mehrparvar

**Affiliations:** ^1^Industrial Diseases Research Center, Shahid Sadoughi University of Medical Sciences, Yazd 89138-14389, Iran; ^2^Department of Radiology, Shahid Sadoughi University of Medical Sciences, Yazd 89138-14389, Iran; ^3^Department of Occupational Medicine, Shahid Sadoughi University of Medical Sciences, Yazd 89138-14389, Iran; ^4^Department of Occupational Health, Shahid Sadoughi University of Medical Sciences, Yazd 89138-14389, Iran; ^5^Shahid Sadoughi University of Medical Sciences, Yazd 89138-14389, Iran; ^6^Department of Radiology, Faculty of Medicine, Shahrekord University of Medical Sciences, Shahrekord 8815713471, Iran

## Abstract

**Introduction:**

Exposure to high intensity noise produced by MRI is a cause for concern. This study was conducted to determine the temporary and permanent effects of exposure to noise created by performing MRI on the hearing threshold of the subjects using conventional and extended high frequency audiometry.

**Methods:**

This semiexperimental study was performed on 35 patients referred to Shahid Rahnemoun Hospital for head and neck MRI due to different clinical conditions. The hearing threshold of patients was measured before, immediately after, and 24 hours after performing 1.5 Tesla MRI using conventional and extended high frequency audiometry. SPSS version 18 was used to compare the mean hearing thresholds before and after MRI using paired T test and repeated measures analysis.

**Results:**

Comparison of auditory thresholds in conventional and extended high frequencies before and immediately after MRI showed a significant shift at 4 KHz (P = 0.008 and P = 0.08 for right and left ears), 6 KHz (P = 0.03 and P = 0.01 for right and left ears), and 14 KHz (P =0.03 and P = 0.31 for right and left ears). However, there was no significant difference between audiometric thresholds before and 24 hours after MRI.

**Conclusion:**

Noise due to 1.5 Tesla MRI can only cause transient threshold shift.

## 1. Introduction

High levels of noise can temporary impair hearing thresholds and cause temporary threshold shift (TTS) which is probably reversed after abstinence from noise exposure. Permanent threshold shift (PTS) occurs after a mechanical injury to hair cells due to exposure to noise [1, 2]. According to Occupational Safety and Health Association (OSHA), permissible exposure limit for noise in an 8-hour work shift is 90 dBA [3].

Magnetic resonance imaging (MRI) is an ever-increasing imaging modality used to detect many lesions in the body. New MRI devices, due to their detailed imaging and three-dimensional measurements, are widely used for central nervous system imaging. MRI device produces noise during imaging which is positively related to the strength of magnetic field (in Tesla) and may affect patient's hearing [4–6]. Studies showed that MRI creates higher levels of noise during last gradient echo pulse sequence [7].

Head and neck MRI especially may affect hearing due to the closeness of the device to the ear. Hearing damage after exposure to MRI noise depends on the frequency and intensity of the noise and duration of the exposure and also the distance between ear and MRI device [8].

Some previous studies have found that MRI may affect hearing by oxidative stress and cochlear hair cells injury [9].

The noise produced by MRI devices is dependent on MRI strength, so that the MRI devices with different powers (0.2 to 3 Tesla) produce varying noise levels from 101 to 131 dBA [10, 11]. The frequency of noise produced by MRI devices is mostly around 4 KHz [4, 11]. According to National Institute for Occupational Safety and Health (NIOSH) guidelines, recommended exposure limit to noise with 120 dBA intensity is 7 s. Radomskij et al. in a study on dogs found that hearing threshold was increased about 2-5 dB after exposure to MRI noise in 50% of dogs [9].

The studies about the effect of MRI noise on hearing thresholds are few and controversial. Some case reports have found MRI as the cause of TTS [5, 12] and PTS [13] in humans. Lim et al. could not find the effect of MRI noise on hearing thresholds in both conventional (500-8000 Hz) and extended high frequencies (10000–14000 Hz). They used 3 Tesla MRI and all patients had used ear plugs during imaging [14]. Jin et al. found that 3 Tesla MRI noise can induce TTS in healthy subjects, even with the use of ear protectors [15]. We could not find a study on 1.5 Tesla MRI which is the most commonly used intensity for head and neck imaging.

This study was performed to define the effect of noise produced by 1.5 Tesla MRI on hearing thresholds in different audiometric frequencies (500-16000 Hz) in patients referred for head and neck MRI.

## 2. Materials and Methods

This was a before-after study performed in Shahid Rahnemoun Hospital. Participants were selected by consecutive sampling from patients referred to MRI center of Shahid Rahnemoun Hospital to perform head and neck MRI with different indications during January till December 2017. An informed consent was obtained from each participant. Patients older than 50 years were not selected and those with moderate hearing loss (hearing threshold higher than 40 dB at each frequency) and conductive hearing loss were excluded from the study after performing baseline audiometry.

MRI device was a Siemens (Avanto, B19, Germany) with magnetic field intensity of 1.5 Tesla. Five steps were performed for each imaging: localizing (10 s), T1 axial (90 s), T2 axial (120 s), T2 trim (180 s), T2 sagittal (90 s), T2 sagittal (90 s), and T2 coronal (90 s) with 1-2-second interval between each protocol. Totally, imaging lasted about 10 minutes for head and 7 minutes for neck.

Pure-tone audiometry (PTA) was done using a diagnostic audiometer (device: Interacoustic AC40, Denmark, headphone: TDH-39 for conventional and Koss R/80 for extended high frequencies, oscillator B70 for bone conduction) by an expert audiologist for each participant at three occasions: (1) 10 minutes before imaging (baseline); (2) during 1 hour after imaging (to detect TTS); and (3) between 24 and 48 hours after imaging (to detect PTS). Hearing threshold for air conduction (AC) and bone conduction (BC) was measured for each frequency in each ear separately. Hearing frequencies which were tested by PTA included 500, 1000, 2000, 3000, 4000, 6000, 8000, 10000, 12000, 14000, and 16000 Hz.

Data were analyzed by SPSS ver. 19 using paired T test and repeated measures analysis.

## 3. Results

Initially 62 patients between 14 and 45 years old were selected. After the first audiometry, 29 patients were excluded due to moderate hearing loss or conductive hearing loss, and at last 33 patients continued the study.

Mean (±SD) of age was 31 (±9.7) years (range: 16-45). Threshold shift was observed after MRI and the highest threshold shift one hour after MRI was observed at 4 KHZ and 14 KHz in conventional and extended high frequency audiometry in both ears.  Table 1 compares hearing threshold of different frequencies before, 1 hour after, and 24 hour after MRI.

Table 2 shows the P value for the comparison of mean hearing thresholds at different frequencies at three occasions (baseline, 1 hour, and 24 hours after MRI) calculated by repeated measures analysis.

## 4. Discussion

Hearing damage due to loud noise can be temporary or permanent. Many individuals experience a TTS after exposure to loud noise which disappears some hours after the termination of exposure; but in some individuals the hearing loss may exist even after several hours of abstinence from exposure to noise. In permanent hearing loss, hair cells of the organ of Corti are damaged due to some mechanisms.

MRI is a widely used imaging method in different disciplines of medicine and due to its advantages, its application is being increased. Exposure to high levels of noise is one of the problems which the patients may experience during imaging and this loud noise may cause TTS or PTS. Wagner et al. found that MRI produces a sound pressure level at the patient's ear between 79.5 to 86.5 dBA and sort-term peaks up to 120 dB [16].

In this study, we examined the effect of noise produced by 1.5 Tesla MRI on hearing status of the patients. We assessed both TTS and PTS at conventional and extended high frequencies. The results showed that TTS was observed at 4, 6, 8, and 14 KHz frequencies, but this threshold shift disappeared after 24 hours, so no PTS was observed.

The results of the studies on hearing loss after MRI are controversial. Most studies have assessed TTS at conventional frequencies [5, 12, 13]. A study showed that those who undergone 0.5 Tesla MRI without wearing hearing conservation devices experienced headache, earache, and tinnitus after imaging [17]. Govindaraju et al. found TTS and tinnitus after exposure to 3 Tesla MRI of spine, but the hearing loss was disappeared after 3 days, and tinnitus remained [5]. These results are in accordance with the results of the present study. Mollasadeghi et al. reported a case of permanent hearing loss after exposure to MRI noise [13].

The results of the present study showed that bilateral hearing loss was observed at 4, 6, and 14 KHz which are the most common frequencies affected by continuous noise in occupational settings [18–20]. Hearing loss at 14 and 16 KHz has been also shown after exposure to MRI noise [20–23].

Radomskij et al. found that 1.5 tesla MRI caused 68% more hearing changes measured by OAEs in patients not wearing ear plugs compared those wearing ear plugs [9]. They did not assess PTS. Lim et al. and Wagner et al. did not find any changes in hearing status in patients undergoing MRI who used hearing protection devices [14, 16].

In the present study, patients had authority to use hearing protection devices and some of them used them. Jin et al. used ABR for assessing the effect of 3 Tesla MRI noise on hearing and found only TTS in the patients who wore hearing protection devices, which was consistent with the results of the present study [15].

## 5. Conclusion

This study showed that noise produced by 1.5 Tesla MRI during head and neck MRI probably causes TTS without permanent hearing threshold shift.

## Figures and Tables

**Table 1:** Comparison of mean of hearing thresholds at different frequencies of both ears before MRI (baseline), 1 hour after MRI (TTS), and 24 hours after MRI (PTS).

Frequency (KHz)	Ear	Mean (SD) of Hearing thresholds (dB)			Comparison between baseline and 1 hr after MRI		Comparison between baseline and 24 hr after MRI	
		Baseline	after MRI					
			1 hr	24 hrs	Mean difference	P-value	Mean difference	P-value
0.5	Right	9.39 (2.07)	9.39 (2.07)	9.39 (2.07)	0	1	0	1
	Left	9.54 (1.92)	9.54 (1.92)	9.54 (1.92)	0	0.31	0	1

1	Right	10.90 (4.23)	11.06 (4.09)	11.06 (4.09)	0.97	0.31	0.16	0.31
	Left	10.15 (4.23)	10.30 (4.13)	10.30 (4.13)	0.30	1	0.15	0.31

2	Right	10.15 (4.4)	10.15 (4.1)	10.15 (4.1)	0.45	0.18	0	1
	Left	11.51 (5.51)	11.21 (5.59)	11.21 (5.59)	0.15	0.15	-0.30	0.31

3	Right	11.36 (4.55)	11.51 (4.41)	11.51 (4.41)	0.60	0.1	0.15	0.31
	Left	11.81 (7.37)	11.81 (7.37)	11.81 (7.37)	0.91	0.70	0	1

4	Right	11.36 (6.76)	11.51 (5.37)	11.51 (5.37)	1.06	0.008	0.15	0.73
	Left	13.03 (7.17)	13.18 (7.16)	13.18 (7.16)	1.06	0.08	0.15	0.31

6	Right	16.96 (7.8)	17.27 (7.71)	17.27 (7.71)	0.91	0.03	0.31	0.37
	Left	18.33 (7.14)	18.48 (7.44)	18.48 (7.44)	1.03	0.01	0.15	0.78

8	Right	16.06 (9.16)	16.21 (7.6)	16.21 (7.6)	1.06	0.19	0.15	0.48
	Left	17.12 (9.01)	17.27 (8.93)	17.27 (8.93)	0.75	0.04	0.15	0.31

10	Right	10.45 (12.7)	10.45 (12.7)	10.45 (12.7)	0	1	0	1
	Left	7.87 (13.23)	7.87 (13.23)	7.87 (13.23)	0.31	0.32	0	1

12	Right	15.45 (15.73)	15.30 (15.6)	15.30 (15.6)	0.45	0.18	-0.15	0.55
	Left	14.09(13.31)	14.24 (13.23)	14.24 (13.23)	0.60	0.32	0.13	0.31

14	Right	16.21 (19.72)	16.36 (19.77)	16.36 (19.77)	1.21	0.03	0.15	0.31
	Left	13.78 (18.49)	14.54 (13.23)	14.54 (13.23)	1.22	0.31	0.76	0.1

16	Right	29.09 (22.51)	29.09 (22.51)	29.09 (22.51)	0.30	0.31	0	1
	Left	26.96 (22.6)	27.12 (22.6)	27.12 (22.6)	0.46	0.1	0.16	0.31

**Table 2:** P value of the comparison of mean hearing thresholds at three occasions (baseline, 1 hour, and 24 hours after MRI).

Frequency	Right ear	Left ear
500	0.983	0.876

1000	0.372	0.226

2000	0.167	0.591

3000	0.114	0.047

4000	0.025	0.005

6000	0.028	0.012

8000	0.194	0.465

10000	0.728	0.374

12000	0.239	0.114

14000	0.026	0.015

16000	0.374	0.096

## Data Availability

The SPSS data used to support the findings of this study are available from the corresponding author upon request.
